# Acetylation of BcHpt Lysine 161 Regulates *Botrytis cinerea* Sensitivity to Fungicides, Multistress Adaptation and Virulence

**DOI:** 10.3389/fmicb.2019.02965

**Published:** 2020-01-08

**Authors:** Qianqian Yang, Limin Song, Zhengang Miao, Meiling Su, Wenxing Liang, Yawen He

**Affiliations:** ^1^Key Lab of Integrated Crop Pest Management of Shandong, College of Plant Health and Medicine, Qingdao Agricultural University, Qingdao, China; ^2^Shandong Province Key Laboratory of Applied Mycology, Qingdao Agricultural University, Qingdao, China; ^3^State Key Laboratory of Microbial Metabolism, Shanghai Jiao Tong University, Shanghai, China

**Keywords:** *Botrytis cinerea*, lysine acetylation, histidine phosphotransfer protein, dicarboximides and demethylation inhibitor sensitivity, osmotic and oxidative stress, virulence

## Abstract

BcHpt is a core element of the high-osmolarity glycerol (HOG) transduction pathway in *Botrytis cinerea*. In contrast to other elements of the pathway, which have been characterized and proven to play important roles in vegetative differentiation, fungicide resistance, the multistress response, and virulence in *B. cinerea*, BcHpt (Histidine-containing phosphotransfer) is essential but uncharacterized in *B. cinerea*. Our previous study reported the first lysine acetylation site (Lys161) in BcHpt. In this study, the functions of this lysine acetylation site in BcHpt were characterized using site-directed mutagenesis. To mimic Lys161 acetylation, we generated the mutant strain ΔBcHPt + BcHpt^K161Q^-GFP, which exhibited a slower growth rate; lower pathogenicity; higher sensitivity to multiple stresses, including osmotic and oxidative stresses, dicarboximides, and demethylation inhibitors (DMIs); and lower BcSak1 phosphorylation levels than wild-type *B. cinerea*. Constitutive acetylation of BcHpt Ly161 apparently inhibits hyphal growth, the multistress response, and sensitivity to fungicides in *B. cinerea.* Moreover, the lysine acetylation site affected phosphorylation of the MAPK BcSak1.

## Introduction

Mitogen-activated protein kinase (MAPK) signaling pathways play important roles in the response of fungal pathogens to various extracellular stresses, including osmotic and oxidative stress (Kruppa and Calderone, 2006). In *Saccharomyces cerevisiae*, the high-osmolarity glycerol (HOG) pathway is one of the most well-characterized MAP kinase modules (Maeda et al., 1995; Posas and Saito, 1997; Barrett and Hoch, 1998; Koretke et al., 2000; Santos and Shiozaki, 2001). In *S. cerevisiae*, the HOG pathway contains two branches (Sln1 and Sho1), which converge on the MAPKK Pbs2 (Hohmann, 2002). The Sln1 branch consists of Ypd1, Skn7, Ssk1, and Ssk2/Ssk22. Sln1 can sense extracellular osmotic conditions and transfer different signals to downstream MAPK by sequential phosphorylation (Posas et al., 1996; Krantz et al., 2006). In many filamentous fungi phosphorylation of Ssk1 triggers activation of the downstream component. The Sho1 branch consists of Sho1, Cdc42, Ste20, Ste50, Ste11, and Pbs2 (Posas and Saito, 1997; O’Rourke and Herskowitz, 1998).

In filamentous fungi, several elements of the HOG pathway have been identified: an osmosensor histidine kinase (BcOs1, or Bcbos1 in *Botrytis cinerea*); a histidine phosphotransfer (Hpt) protein (Ypd1 in *S. cerevisiae*, BcHpt in *B. cinerea*); two response regulators (Ssk1 in *S. cerevisiae*, BRrg1 in *B. cinerea*); and downstream MAPK cascades containing (Ssk2/Ssk22 in *S. cerevisiae*, BcOs4 in *B. cinerea*; Pbs2 in *S. cerevisiae*, BcOs5 in *B. cinerea* and Hog1 in *S. cerevisiae*, BcSak1/BcOs2 in *B. cinerea*) (Viaud et al., 2006; Noguchi et al., 2007; Segmüller et al., 2007; Liu et al., 2008; Yan et al., 2010, 2011; Fillinger et al., 2012; Yang et al., 2012). Jiang et al. (2018) reviewed MAPK signaling in plant pathogenic fungi. The HOG pathway plays a species-specific and tissue-specific role in fungal virulence (Dixon et al., 1999; Mehrabi et al., 2006; Moriwaki et al., 2006; Jiang et al., 2018). In addition, the HOG pathway is important for mediating osmotic and oxidative stress responses and fungicide (phenylpyrrole and dicarboximide) sensitivity. Many researchers have suggested that the HOG pathway is the target of fludioxonil and fenpiclonil, which are phenylpyrrole fungicides (Jespers et al., 1993; Zhang et al., 2002; Fujimura et al., 2003; Motoyama et al., 2005; Noriyuki et al., 2007; Motoyama et al., 2008; Jespers and Waard, 2010). However, the role of this pathway in responses to other (e.g., cell wall) stresses varies among fungi (Jiang et al., 2018). In addition, the HOG pathway is also involved in the species-specific regulation of hyphal growth and development (Zhang et al., 2002; Jones et al., 2007; Vitalini et al., 2007).

The role of HPt proteins has been characterized in several fungi (Aoyama et al., 2000; Vargas-Pérez et al., 2007; Lee et al., 2011; Mavrianos et al., 2014; Jacob et al., 2015). Numerous fungi contain only one HPt protein, and it seems to integrate all signals from different sensor histidine kinases. Thus, Hpt is essential in many fungi, including *S. cerevisiae*, *N. crassa* and *Aspergillus nidulans* (Maeda et al., 1994; Banno et al., 2007; Furukawa et al., 2010). Some researchers have considered this protein a suitable target for novel antifungal drugs (Fassler and West, 2013).

*Botrytis cinerea* causes gray mold on over 400 plant species, leading to extreme financial losses worldwide (Williamson et al., 2007; Dean et al., 2012; Fillinger and Elad, 2016). Although most core elements are well characterized in *B. cinerea*, the function of BcHpt remains unclear. Our previous proteomics study in *B. cinerea* identified one acetylation site, Lys161, in BcHpt (Lv et al., 2016); this acetylation site was the first to be reported in BcHpt. To determine the role of lysine acetylation in BcHpt, we characterized Lys161 of BcHpt in *B. cinerea* using site-directed mutagenesis.

## Materials and Methods

### Strains and Culture Conditions

The standard reference strain B05.10 of *B. cinerea* Pers. Fr. [*Botrytis fuckeliana* (de Bary) Whetzel] was isolated from *Vitis vinifera* (Quidde et al., 1999). All *B. cinerea* strains used in this study were grown on potato dextrose agar (PDA: 200 g of potato, 20 g of dextrose, 20 g of agar, and 1 L of water).

Conidium and sclerotium formation was assessed after ten days or 4 weeks of incubation on PDA medium. Growth assays were conducted under different stress conditions, and the percentage of mycelial radial growth inhibition (RGI) was measured after 3 days of incubation on PDA as previously described (Yang et al., 2018).

### Generation of the BcHpt Mutant Strains by Site-Directed Mutagenesis

The primers used in this study are listed in the Supplementary Table S1. Since the deletion of Hpt1 in *B. cinerea* is lethal (data not shown), generation of the BcHpt mutant strains was carried out by site-directed mutagenesis using the following protocol: First, the primers which contain the mutated site were designed and listed in Supplementary Table S1 (BcHpt-GFP-F + BcHpt-Q-R and BcHpt-Q-F + BcHpt-GFP-R for the BcHpt-Q-up and BcHpt-Q-down fragments, respectively; BcHpt-GFP-F + BcHpt-R-R and BcHpt-R-F + BcHpt-GFP-R for the BcHpt-R-up and BcHpt-R-down fragments, respectively) and used to amplify the BcHpt gene. Fusion PCR (BcHpt-Q-up and BcHpt-Q-down fragments; BcHpt-R-up and BcHpt-R-down fragments) was employed using BcHpt-GFP-F + BcHpt-GFP-R (Supplementary Table S1) to amplify the BcHpt^K161Q^ and BcHpt^K161R^ sequences (Yu et al., 2004). The resulting sequences were cotransformed with XhoI-digested pYF11 plasmid into the yeast strain XK1-25 to generate BcHpt^K161Q/R/K^-GFP fusion vectors (Bruno et al., 2004). The resulting vectors: BcHpt^K161Q^-GFP-pYF11, BcHpt^K161R^-GFP-pYF11, and BcHpt^K161K^-GFP-pYF11, were transformed into the B05.10 strain using protoplast formation and transformation of *B. cinerea* (Gronover et al., 2001; Jiang et al., 2011), and the resulting transformants (named B05.10 + BcHpt^K16^1Q-GFP, B05.10 + BcHptK161R-GFP, and B05.10 + BcHptK161K-GFP) were confirmed by PCR (GFP-F and GFP-R for detection of *GFP* gene), sequencing (BcHpt-SE for detection of site mutation) and Western blotting (using an anti-GFP antibody to confirm the expression of BcHpt^K161Q/R/^-GFP).

Subsequently, the native BcHpt locus in the resulting transformants was deleted by a homologous recombination strategy to generate the mutant ΔBcHPt + BcHPt^K161Q^, ΔBcHPt + BcHpt^K161R^, and ΔBcHPt + BcHpt^K161K^ strains (Supplementary Figure S1). The gene deletion vector was constructed by inserting two flanking sequences (BcHpt-up-F and BcHpt-up-R for BcHpt-up fragment; BcHpt-down-up and BcHpt-down-R for BcHpt-down fragment) of the BcHPT gene into two sides of the HPH (hygromycin resistance) gene in the pBS-HPH1 vector. The resulting vector, pBS-BcHPT-Del, was transformed into B05.10 + BcHpt^K161Q^-GFP, B05.10 + BcHpt^K161R^-GFP, and B05.10 + BcHpt^K161K^-GFP strains using protoplast formation and transformation of *B. cinerea*. The gene deletion mutants were identified by PCR assays (BcHpt-out-F + BcHpt-out-R), and further confirmed by a Southern blot assay. The probe used in the Southern blot analysis was the BcHPT downstream fragment, which was amplified using BcHpt-down-F and BcHpt-down-R. DNA extracted from the mutants was digested with *Bam*HI. In total of six ΔBcHPt + BcHpt^K161Q^-GFP, four ΔBcHPt + BcHpt^K161R^-GFP, and three ΔBcHPt + BcHpt^K161K^-GFP were obtained, and showed similar phenotypes. Single spore mutants were isolated and transferred to selection medium.

### Nucleic Acid Manipulations and qRT-PCR

Genomic DNA was extracted from *B. cinerea* as previously described (McDonald and Martinez, 1990). Plasmid miniprep purification kits (BioDev Co., Beijing, China) were used to purify plasmid DNA.

The expression levels of BcHpt were tested by qRT-PCR using the 2^–ΔΔCt^ method (Livak and Schmittgen, 2001). Mycelia of the mutants were harvested after 2 days incubation in potato dextrose broth (PDB) in a shaker. RNA was extracted using a protocol described previously (Yang et al., 2018). Reverse transcription was carried out using Revert Aid H Minus First Strand cDNA Synthesis kits (Fermentas Life Sciences, Burlington, Canada). qRT-PCR was conducted using TAKARA SYBR Premix Ex Taq (TAKARA Bio Inc., Dalian, China) with the listed primers (Supplementary Table S1). β-tubulin gene was amplified as a reference. Three biological replicates were used for each sample.

### Pathogenicity and Infection-Related Morphogenesis Assays

Pathogenicity testing was performed as previously described (Yang et al., 2013, 2018). An infection-related morphogenesis assay was performed on onion epidermis as previously described (Doehlemann et al., 2006; Viaud et al., 2006).

### Western Blot Assay

To confirm the expression of the fusion proteins BcHpt^K161Q^-GFP, BcHpt^K161R^-GFP and BcHpt^K161K^-GFP, Western blotting was carried out using an anti-GFP antibody (Beyotime, Shanghai, China) and anti-acetyl lysine mouse mAb (clone Kac-01, PTM-101) (PTM Biolabs, Hangzhou, China). Conidia were harvested from 10-day old cultures. The mycelia of the mutants were grown in yeast extract peptone dextrose (YEPD) at 25°C for 2 days in a shaker. After the cultures were treated with 20 mM H_2_O_2_, 0.2 mg/ml congo red, 1 M NaCl and 1 μg/ml iprodione for 2 h, mycelia were harvested. Protein extraction was carried out as previously described (Gu et al., 2015). The expression of BcSak1 and phosphorylated BcSak1 was examined by using an anti-Hog1 antibody (Santa Cruz Biotechnology, CA, United States) and an antibody against dually phosphorylated p38 (Thr180/Tyr182) (Cell Signaling Technology, MA, United States).

## Results

### Generation of the BcHpt Mutants by Site-Directed Mutagenesis

**FIGURE 1:**
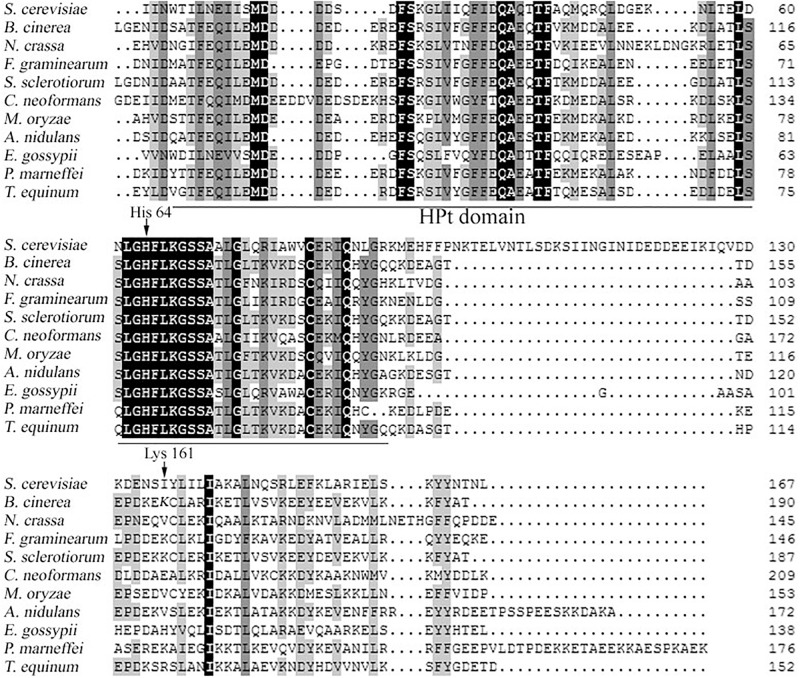
Multiple sequence alignment of the HPt domain of several fungal Ypd1 orthologs. The genome database accession numbers are as follows: *Saccharomyces cerevisiae* NP_010046.1, *Botrytis cinerea* XP_024552997.1, *N. crassa* XP_011393451.1, *F. graminearum* XP_011321244.1, *S. sclerotiorum* SS1G_10394, *Cryptococcus neoformans* XP_568444.1, *Magnaporthe oryzae* XP_003715375.1, *A. nidulans* XP_659609.1, *Eremothecium gossypii* AAS50589.1, *P. marneffei* XP_002151180.1, and *Trichophyton equinum* EGE05000.1. BoxShade was used to highlight identical (black shading) or similar (gray shading) amino acids. The Hpt domain is underlined, and the His64 and Lys161 positions are indicated with arrows.

**FIGURE 2:**
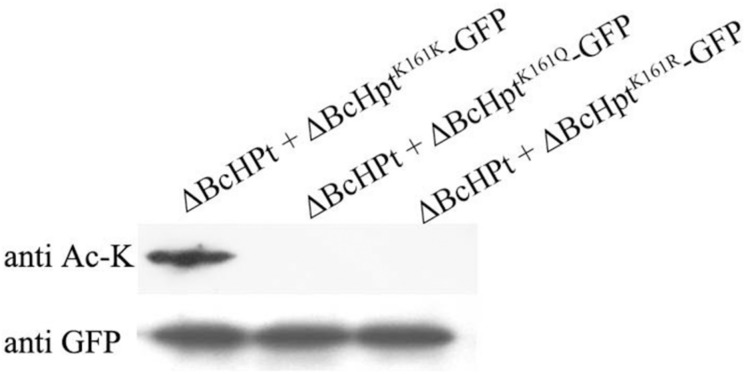
Acetylation levels of BcHPt in ΔBcHpt + BcHpt^K161K^-GFP, ΔBcHpt + BcHpt^K161Q^-GFP, and ΔBcHpt + BcHpt^K161R^-GFP. BcHpt^K161Q^-GFP, BcHpt^K161R^-GFP, and BcHpt^K161K^-GFP were transformed into *B. cinerea*, and BcHpt-GFP was pulled down in protein extracts from the mutants using anti-GFP antibody agarose beads. The acetylation and total protein levels were determined using an anti-acetyl lysine antibody and anti-GFP antibody, respectively.

In our previous study, we obtained more than 800 ectopic mutants without deletion mutant, thus we believe BcHpt is essential (data not shown). Our previous proteomics study identified one acetylation site, Lys161, in BcHpt (Lv et al., 2016). The Lys161 acetylation site in BcHpt is conserved in 4 of the 11 fungal species analyzed (*B. cinerea*, *Sclerotinia sclerotiorum*, *Fusarium Graminearum*, and *Penicillium marneffei*) (Figure 1). In *B. cinerea*, Lys 161 is not inside the HPt domain of BcHpt (66–146) (Figure 1). Deletion of Hpt in *B. cinerea* and several other fungi was reported to be lethal (Maeda et al., 1994; Banno et al., 2007; Furukawa et al., 2010). Thus, to verify the function of the lysine acetylation site in BcHpt, BcHpt containing either the K161Q or K161R mutation was introduced into the wild-type strain B05.10. Glutamine (Q) and arginine (R) mimic acetylated and unacetylated lysine, respectively (Schwer et al., 2006; Li et al., 2007). In addition, BcHpt-GFP without any point mutations was used as a control. The resulting strains (B05.10 + BcHpt^K161Q^-GFP, B05.10 + BcHpt^K161R^-GFP, and B05.10 + BcHpt^K161K^-GFP) were confirmed by PCR, sequencing and Western blotting using an anti-GFP antibody. Then, the native BcHpt locus was deleted in the transformants using a homologous recombination strategy to generate the mutant ΔBcHPt + BcHpt^K161Q^-GFP, ΔBcHPt + BcHpt^K161R^-GFP, and ΔBcHPt + BcHpt^K161K^-GFP strains. The acetylation levels of the purified BcHpt^K161Q/R/K^-GFP proteins were determined using a pan anti-acetyl lysine antibody. Western blotting showed that ΔBcHPt + BcHpt^K161Q^-GFP and ΔBcHPt + BcHpt^K161R^-GFP were not acetylated but that ΔBcHPt + BcHpt^K161K^-GFP was, indicating that Lys161 is the only acetylation site in BcHpt (Figure 2). In addition, as shown in Supplementary Figure S2, the expression levels of BcHPT in ΔBcHPt + BcHpt^K161Q^-GFP, ΔBcHPt + BcHpt^K161R^-GFP, and ΔBcHPt + BcHpt^K161K^-GFP were similar to that in B05.10. Thus the phenotypic changes were not due to differences in the expression levels.

### Acetylation of BcHpt1-Lys161 Has Negative Effects on Hyphal Growth but Not on Conidium and Sclerotium Formation in *B. cinerea*

**FIGURE 3:**
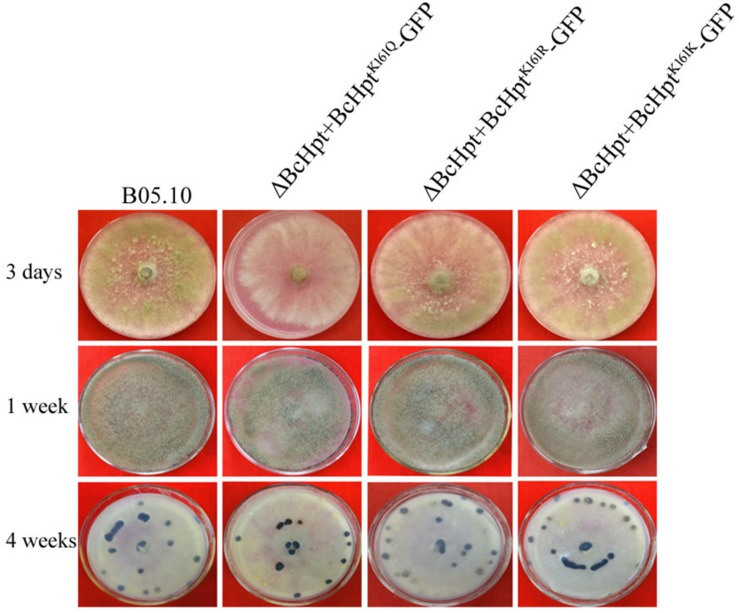
BcHpt Lys161 is involved in hyphal growth but not in conidium and sclerotium formation in *B. cinerea*. Morphology of B05.10, ΔBcHpt + BcHpt^K161Q^-GFP, ΔBcHpt + BcHpt^K161R^-GFP, and ΔBcHpt + BcHpt^K161K^-GFP on PDA medium after 3 days, 1 week and 4 weeks of incubation.

The mycelial growth rate of ΔBcHPt + BcHpt^K161K^-GFP and ΔBcHPt + BcHpt^K161R^-GFP was similar to that of the wild-type parent B05.10, while ΔBcHPt + BcHpt^K161Q^-GFP showed minor growth retardation compared to B05.10 (Figure 3 and Supplementary Figure S3). After 3 days of incubation, the colony diameters of ΔBcHPt + BcHpt^K161Q^ were reduced by 15%. However, the formation of conidia and sclerotia in the three mutants was similar to that in B05.10 after one or 4 weeks of incubation on PDA (Figure 3). These results indicate that acetylation of BcHpt1 Lys161 down-regulates hyphal growth but does not affect conidium and sclerotium formation in *B. cinerea*.

### BcHpt Lys161 Is Involved in the Fungicide and Stress Sensitivity of *B. cinerea*

**FIGURE 4:**
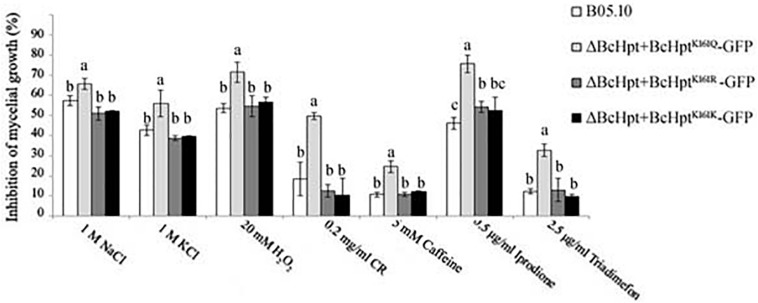
Sensitivity of B05.10, ΔBcHpt + BcHpt^K161Q^-GFP, ΔBcHpt + BcHpt^K161R^-GFP, and ΔBcHpt + BcHpt^K161K^-GFP to multiple stresses. Inhibition of mycelial growth in the treated strains compared with the growth of the untreated control strains. The bars denote the standard errors from three experiments, and statistical tests were carried out using Tukey’s test for multiple comparisons. Values on the bars followed by different letters are significantly different at *P* = 0.05.

BcHpt is a core element of the HOG pathway, which can be activated by various stresses, including oxidative and osmotic stresses, fungicides and hormones; thus, the sensitivity of the mutants to these stresses was investigated. As shown in Figure 4 and Supplementary Figure S4, compared to the B05.10, ΔBcHPt + BcHpt^K161R^-GFP, and ΔBcHPt + BcHpt^K161K^-GFP strains, ΔBcHPt + BcHpt^K161Q^-GFP exhibited increased sensitivity to iprodione and triadimefon, which belong to dicarboximides and ergosterol biosynthesis inhibitors (EBIs), respectively. Furthermore, ΔBcHPt + BcHpt^K161Q^-GFP was also more sensitive to osmotic (NaCl and KCl), oxidative (H_2_O_2_), and cell wall stress (Congo red and caffeine) than the wild-type strain. These results indicate that constitutive acetylation of BcHpt Lys161 increases sensitivity of *B. cinerea* to fungicides (dicarboximides and DMIs) exposure, and multiple stresses, including oxidative, osmotic, and cell wall stresses.

### Regulation of BcSak1 Phosphorylation by BcHpt Lys161

**FIGURE 5:**
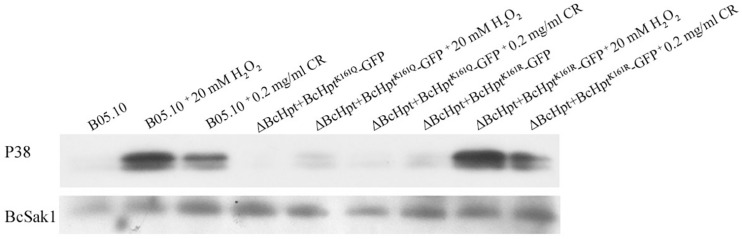
BcHpt Lys161 affects the phosphorylation levels of BcSak1. Comparison of BcSak1 phosphorylation in B05.10, ΔBcHpt + BcHpt^K161Q^-GFP and ΔBcHpt + BcHpt^K161R^-GFP. Phosphorylated and total BcSak1 proteins were detected using anti-phosphorylated p38 (Thr180/Tyr182) and anti-Hog1 antibodies, respectively.

Phosphorylation of BcSak1 indicates the activation of the HOG pathway in *B. cinerea*; thus, we analyzed the effects of BcHpt Lys161 acetylation on the phosphorylation of BcSak1. Two bands were detected for the phosphorylated Sak1, consistent with the findings of previous studies (Liu et al., 2008; Yang et al., 2013). Western blot analysis showed that BcSak1 phosphorylation was strongly increased in response to 20 mM H_2_O_2_ and 0.2 mg/ml congo red in B05.10 and ΔBcHPt + BcHpt^K161R^-GFP but not in ΔBcHPt + BcHpt^K161Q^-GFP (Figure 5). In addition, in response to 1 M NaCl and 1 μg/ml iprodione, BcSak1 phosphorylation was strongly increased in B05.10 but not in ΔBcHPt + BcHpt^K161Q–^GFP (Supplementary Figure S5). These results indicate that constitutive acetylation of BcHpt Lys161 affects the phosphorylation level of BcSak1 especially under activating conditions.

### Acetylation of BcHpt in Response to Various Stresses

**FIGURE 6:**
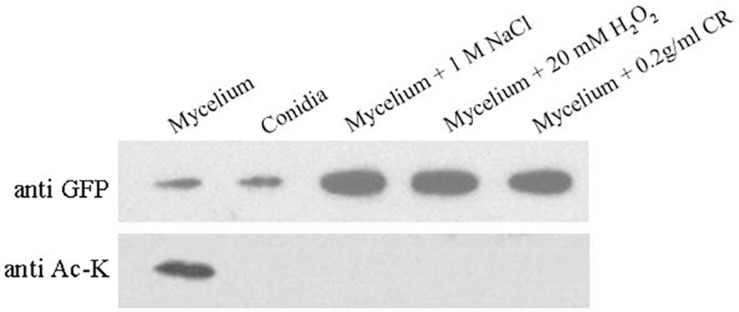
Acetylation of BcHpt in response to various stresses. Comparison of BcHpt acetylation in ΔBcHpt + BcHpt^K161K^-GFP. Acetylated and total BcHpt proteins were detected using anti-Ac-K and anti-GFP antibodies, respectively.

Figure 5 indicates that BcHpt Lys161 affects the phosphorylation level of BcSak1 in response to various stresses; thus, it would be interesting to know whether the acetylation status of Lys161 can change in response to various stresses. As shown in Figure 6, BcHpt acetylation could be detected in the mycelium, but not in conidia. Furthermore, in response to osmotic (NaCl), oxidative (H_2_O_2_), and cell wall (Congo red) stresses, the acetylation levels of BcHpt were significantly decreased.

### Constitutive Acetylation of BcHpt Lys161 Affects the Virulence of *B. cinerea*

**FIGURE 7:**
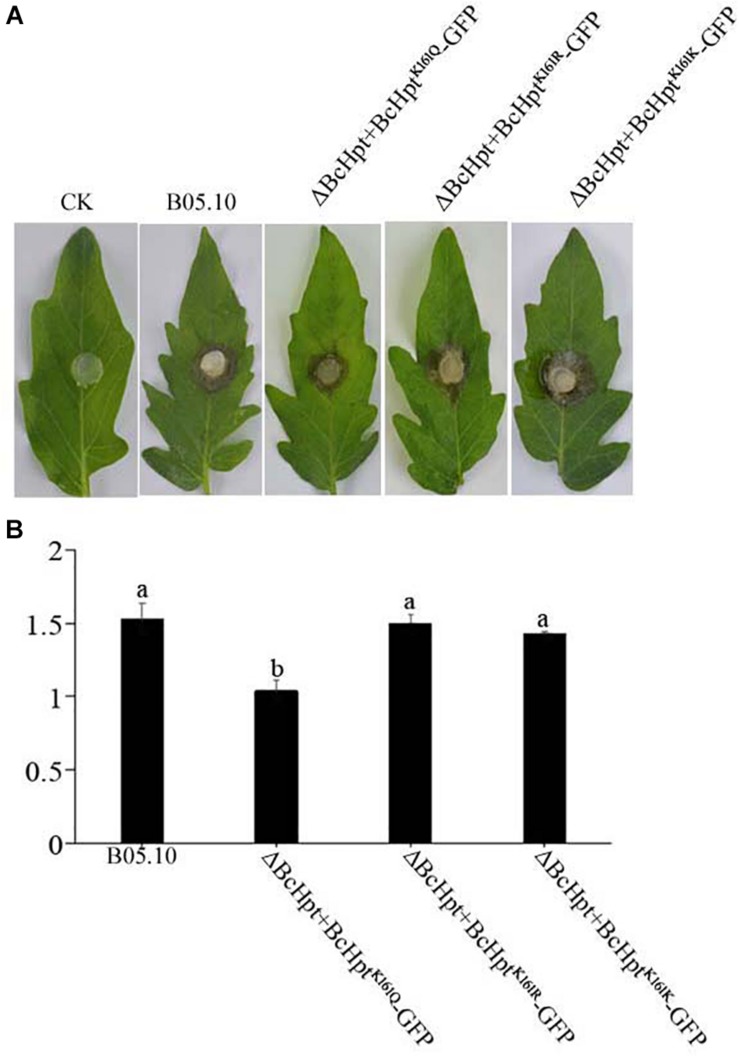
Constitutive acetylation of BcHpt Lys161 affects the virulence of *B. cinerea*. Virulence tests were performed on tomato leaves following inoculation with B05.10, ΔBcHpt + BcHpt^K161Q^-GFP, ΔBcHpt + BcHpt^K161R^-GFP, and ΔBcHpt + BcHpt^K161K^-GFP or agar plugs as negative controls (CK). **(A)** Disease symptoms caused by B05.10, ΔBcHpt + BcHpt^K161Q^-GFP, ΔBcHpt + BcHpt^K161R^-GFP, and ΔBcHpt + BcHpt^K161K^-GFP on wounded tomato leaves 3 days after inoculation. **(B)** Diameter of disease lesions on tomato leaves after 3 days of incubation. The bars denote the standard errors of four replicates. Statistical tests were carried out using Tukey’s test for multiple comparisons, and values on the bars followed by different letters are significantly different at *P* = 0.05.

**FIGURE 8:**
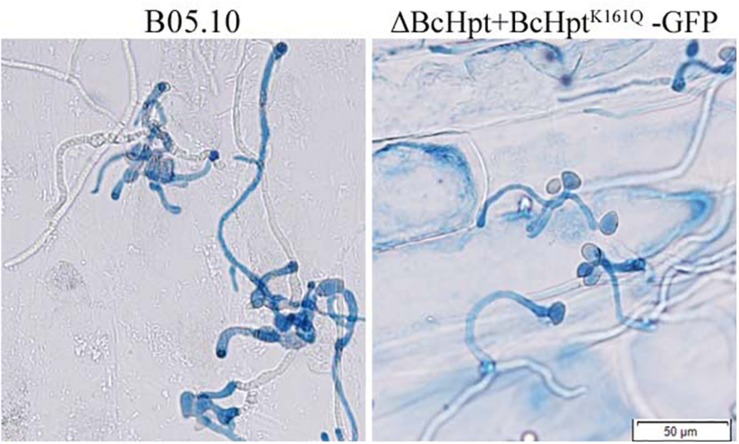
Onion epidermis penetration assay with B05.10 and ΔBcHpt + BcHpt^K161Q^-GFP. Conidia of B05.10 and ΔBcHpt + BcHpt^K161Q^-GFP were incubated with onion epidermis for 15 h before the cultures were photographed.

Since several components of the HOG pathway are involved in the virulence of *B. cinerea*, we also performed an infection test on tomato leaves to test the influence of BcHpt Lys161 on *B. cinerea* pathogenicity. As shown in Figure 7, 3 days after inoculation, ΔBcHPt + BcHpt^K161Q^-GFP caused smaller (approximately 32.3%) lesions on tomato leaves than B05.10, ΔBcHPt + BcHpt^K161R^-GFP and ΔBcHPt + BcHpt^K161K^-GFP (Figure 7). The pathogenicity defects of ΔBcHPt + BcHpt^K161Q^-GFP were analyzed in detail using onion epidermis penetration assays. However, the penetration efficiency of ΔBcHPt + BcHpt^K161Q^-GFP was similar to that of B05.10 (Figure 8). The decreased virulence of ΔBcHPt + BcHpt^K161Q^-GFP was likely due to its minor growth reduction, and increased sensitivity to osmotic and oxidative stresses.

## Discussion

Fungal HOG pathways play various roles in cells, including in osmotic, oxidative and other (e.g., cell wall) stress responses; fungicide (phenylpyrrole and dicarboximide) sensitivity; hyphal growth and development; virulence; sexual and asexual development; secondary metabolite production; and dimorphic phase transitions (Li et al., 2010; Jiang et al., 2018). HPt is essential in *S. cerevisiae*, *N. crassa*, *A. nidulans*, and *B. cinerea* (Maeda et al., 1994; Banno et al., 2007; Furukawa et al., 2010), but the function of BcHPt in *B. cinerea* remains unclear. However, we identified one acetylation site in BcHpt in our previous proteomics studies (Lv et al., 2016). To determine the role of lysine acetylation in BcHpt, we characterized Lys161 of BcHpt in *B. cinerea* by site directed mutagenesis. Since deletion of BcHPt is lethal, but ΔBcHPt + BcHpt^K161Q^-GFP could be produced, acetylation at residue 161 is relevant for some but not all activities of the HPt protein. The regulatory mechanisms of this residue in the HPt protein remain to be further discovered.

To further characterize the function of BcHpt Lys161, we analyzed the phosphorylation levels of BcSak1 in the mutants. Under stresses, the phosphorylation levels of BcSak1 in the ΔBcHPt + BcHpt^K161Q^-GFP mutant were significantly lower than that in the wild-type strain (Figure 5), indicating that acetylation of BcHpt Lys161 affects the phosphorylation level of BcSak1. The HPt domain of *S. cerevisiae* Ypd1 contains a four-helix bundle (αB-αC-αD-αG) as a core structure. In addition, His64 is predicted to play important roles in the phosphoryl transfer activity of Ypd1. The involved residues are conserved in many organisms and have been functionally characterized (Janiak and West, 2000; Fassler and West, 2013). In *B. cinerea*, this residue is His120, and the acetylation site is Lys161, which is not inside the HPt domain of BcHpt (66–146) (Figure 1).

ΔBcHPt + BcHpt^K161Q^-GFP exhibited increased sensitivity to multiple stresses, including fungicides (dicarboximides and DMIs), oxidative, osmotic and cell wall stresses (Figure 3). However, its phenotype under oxidative stress is minor and very different from that observed for a BcSak1 deletion strain (Segmüller et al., 2007). It is reasonable since deletion of BcSak1 led to a complete loss of phosphorylated BcSak1; however, ΔBcHPt + BcHpt^K161Q^-GFP, still has a relatively small amount of phosphorylated BcSak1. In addition, BcSak1 deletion mutants, but not ΔBcHPt + BcHpt^K161Q^-GFP, showed conidium and sclerotium formation changes compared to wild type strain (Segmüller et al., 2007). This is likely because, unlike BcSak1 deletion mutants, ΔBcHPt + BcHpt^K161Q^-GFP still has a low level of phosphorylated BcSak1.

ΔBcHPt + BcHpt^K161Q^-GFP also showed increased sensitivity to triadimefon, which is an ergosterol biosynthesis inhibitor. This phenotype was consistent with those of BRrg1 and BcSkn7 (Yan et al., 2011; Yang et al., 2015). Considering phosphorylation levels of BcSak1 in three mutants (ΔBcHPt + BcHpt^K161Q^-GFP, ΔBRrg1, and ΔBcSkn7) were lower than that in the wild-type strain (Yan et al., 2011; Yang et al., 2015), the triadimefon sensitivity was most likely a consequence of lower phosphorylation levels of BcSak1. However, the mechanisms of HOG pathway involvement in the response to an impaired ergosterol biosynthesis in *B. cinerea* have not been addressed so far.

Acetylation of BcHpt cannot be detected in response to osmotic (NaCl), oxidative (H_2_O_2_), and cell wall (Congo red) stresses, while the phosphorylation levels of BcSak1 were upregulated. These results indicate that acetylation could affect the phosphoryl transfer activity of BcHpt. Since acetylation and phosphorylation are both common post-translational protein modifications, further exploring the regulatory mechanism mediating the acetylation and phosphorylation of BcHpt would be of interest. Moreover, as a cell wall stressor, Congo red could also activate the HOG pathway (Figure 5) and cause deacetylation of BcHpt (Figure 6), which provides further evidence of the cross talk between the HOG and the CWI pathway.

*Saccharomyces cerevisiae* does not contain such a Lys residue, and only 4 out of 11 fungal species analyzed (*B. cinerea*, *Sclerotinia sclerotiorum*, *F. graminearum*, and *P. marneffei*) possess the Lys residue. These four fungal species belonged to *Pezizomycotina*, however, other *Pezizomycotina* fungi (*N. crassa* and *Magnaporthe oryzae*) do not have this residue. Acetylation of this site may impact the phosphorylation of BcSak1 in response to the above cited stresses, which means that the signal transduction is interrupted. Thus, our hypothesis is that acetylation/deacetylation of BcHpt plays important roles in its signal transduction function in the four cited species. As this residue is not conserved among fungal species, further studies are required to investigate signal transduction in the remaining species.

## Conclusion

In conclusion, the acetylation of BcHpt Lys161 plays a significant role in the hyphal growth, osmotic and oxidative stress responses, and dicarboximide and DMI sensitivities of *B. cinerea*. BcHpt is deacetylation in response to osmotic, oxidative, and cell wall stresses. Constitutive acetylation of Lys161 interrupted signal transduction of the HOG pathway, which led to lower phosphorylation levels of BcSak1. The regulatory mechanism mediating these two common post-translational protein modifications requires further study.

## Data Availability Statement

The raw data supporting the conclusions of this artilcle will be made available by the authors, without undue reservation, to any qualified researcher.

## Author Contributions

QY and WL generated the hypothesis and planned the experiments. QY, LS, ZM, and MS performed the experiments. QY, WL, and YH wrote the manuscript. All other authors provided comments on the manuscript.

## Conflict of Interest

The authors declare that the research was conducted in the absence of any commercial or financial relationships that could be construed as a potential conflict of interest.

## Supplementary Material

FIGURE S1 Generation strategy of the BcHpt mutant strains of *B. cinerea*. The hygromycin-resistant cassette (HPH) is denoted by the large gray box, GFP is denoted by the large white box and the neomycin-resistant cassette (NEO) is denoted by the large shadowed area. The vectors: BcHpt^K161K^-GFP- pYF11, BcHpt^K161R^-GFP- pYF11, and BcHpt^K161Q^-GFP- pYF11 (BcHpt^K161K/R/Q^-GFP-pYF11), which contain the site-directed mutagenesis were transformed into the B05.10 strain. The resulting transformants were named B05.10 + BcHpt^K161Q^-GFP, B05.10 + BcHpt^K161R^-GFP, and B05.10 + BcHpt^K161K^-GFP (B05.10 + BcHpt^K161K/R/Q^-GFP), respectively. Subsequently, the native BcHpt locus in the resulting transformants was deleted by a homologous recombination strategy to generate the mutant ΔBcHpt + BcHpt^K161Q^, ΔBcHpt + BcHpt^K161R^, and ΔBcHpt + BcHpt^K161K^ (ΔBcHpt + BcHpt^K161K/R/Q^) strains. Primer (codes 1–17) binding sites are indicated by arrows.

FIGURE S2 Relative expression levels of BcHpt in B05.10, ΔBcHpt + BcHpt^K161Q^-GFP, ΔBcHpt + BcHpt^K161R^-GFP, and ΔBcHpt + BcHpt^K161K^-GFP. Mycelia were incubated in PDB for 2 days. The bars denote the standard errors from three experiments, and statistical tests were carried out using Tukey’s test for multiple comparisons. Values on the bars followed by different letters are significantly different at *P* = 0.05.

FIGURE S3 Morphology of B05.10 and two additional K161Q mutants: ΔBcHpt + BcHpt^K161Q^-GFP-2, and ΔBcHpt + BcHpt^K161Q^-GFP-3 on PDA medium after 3 days of incubation.

FIGURE S4 Sensitivity of B05.10, ΔBcHpt + BcHpt^K161Q^-GFP, ΔBcHpt + BcHpt^K161R^-GFP, and ΔBcHpt + BcHpt^K161K^-GFP grown on PDA containing different compounds at the concentrations noted in the figure. The cultures were photographed after 48 h of incubation at 25°C.

FIGURE S5 Comparison of BcSak1 phosphorylation in B05.10 and ΔBcHpt + BcHpt^K161Q^-GFP in response to 1 M NaCl and 1 μg/ml iprodione.

TABLE S1 Primers used in the study.
