# Supplementary figures and images for: Acetylation of BcHpt Lysine 161 Regulates Botrytis cinerea Sensitivity to Fungicides, Multistress Adaptation and Virulence
Source: Front Microbiol. 2020 Jan 8;10:2965. doi: 10.3389/fmicb.2019.02965 (PMC6960119; doi:10.3389/fmicb.2019.02965)

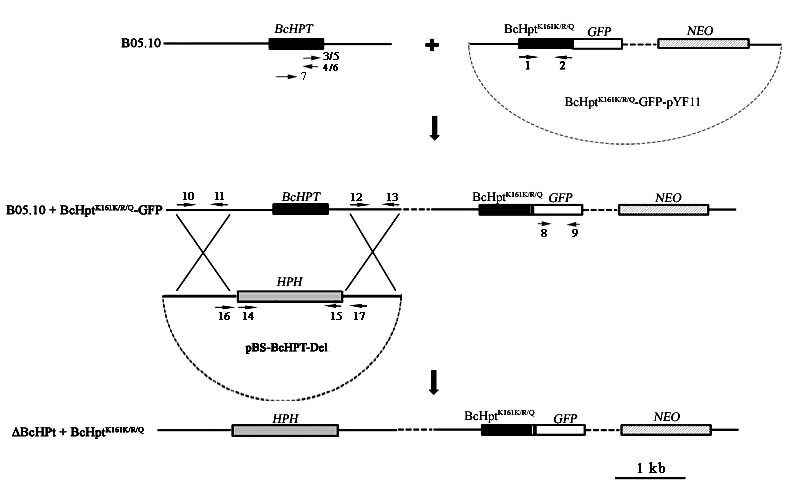

Supplement: FIGURE S1 — Generation strategy of the BcHpt mutant strains of B. cinerea. The hygromycin-resistant cassette (HPH) is denoted by the large gray box, GFP is denoted by the large white box and the neomycin-resistant cassette (NEO) is denoted by the large shadowed area. The vectors: BcHptK161K-GFP- pYF11, BcHptK161R-GFP- pYF11, and BcHptK161Q-GFP- pYF11 (BcHptK161K/R/Q-GFP-pYF11), which contain the site-directed mutagenesis were transformed into the B05.10 strain. The resulting transformants were named B05.10 + BcHptK161Q-GFP, B05.10 + BcHptK161R-GFP, and B05.10 + BcHptK161K-GFP (B05.10 + BcHptK161K/R/Q-GFP), respectively. Subsequently, the native BcHpt locus in the resulting transformants was deleted by a homologous recombination strategy to generate the mutant ΔBcHpt + BcHptK161Q, ΔBcHpt + BcHptK161R, and ΔBcHpt + BcHptK161K (ΔBcHpt + BcHptK161K/R/Q) strains. Primer (codes 1–17) binding sites are indicated by arrows. [file Image_1.JPEG]

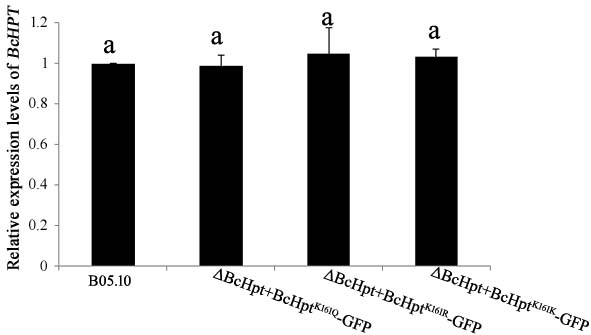

Supplement: FIGURE S2 — Relative expression levels of BcHpt in B05.10, ΔBcHpt + BcHptK161Q-GFP, ΔBcHpt + BcHptK161R-GFP, and ΔBcHpt + BcHptK161K-GFP. Mycelia were incubated in PDB for 2 days. The bars denote the standard errors from three experiments, and statistical tests were carried out using Tukey’s test for multiple comparisons. Values on the bars followed by different letters are significantly different at P = 0.05. [file Image_2.JPEG]

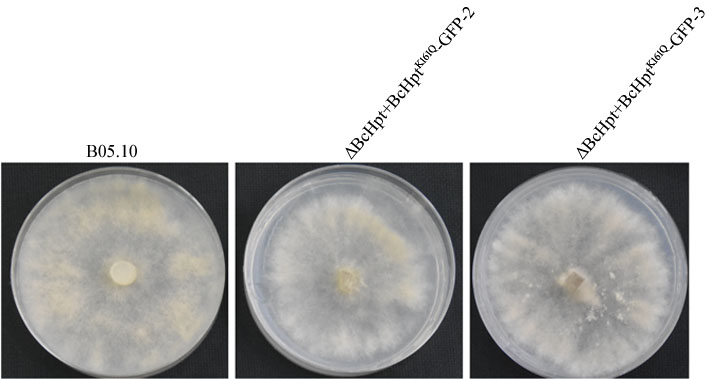

Supplement: FIGURE S3 — Morphology of B05.10 and two additional K161Q mutants: ΔBcHpt + BcHptK161Q-GFP-2, and ΔBcHpt + BcHptK161Q-GFP-3 on PDA medium after 3 days of incubation. [file Image_3.JPEG]

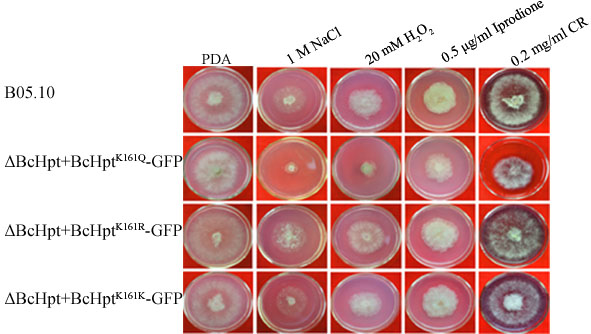

Supplement: FIGURE S4 — Sensitivity of B05.10, ΔBcHpt + BcHptK161Q-GFP, ΔBcHpt + BcHptK161R-GFP, and ΔBcHpt + BcHptK161K-GFP grown on PDA containing different compounds at the concentrations noted in the figure. The cultures were photographed after 48 h of incubation at 25°C. [file Image_4.JPEG]

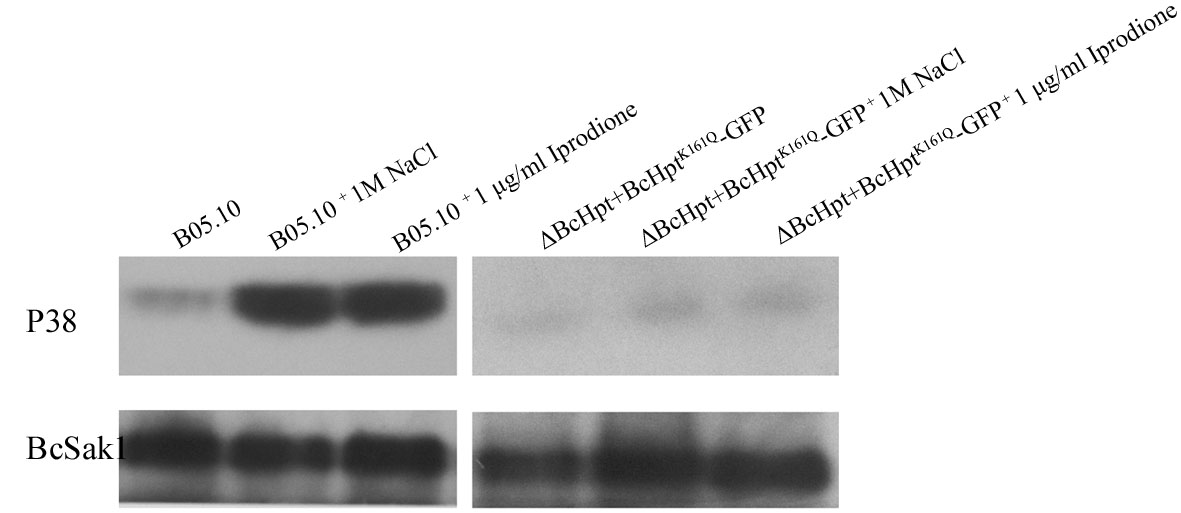

Supplement: FIGURE S5 — Comparison of BcSak1 phosphorylation in B05.10 and ΔBcHpt + BcHptK161Q-GFP in response to 1 M NaCl and 1 μg/ml iprodione. [file Image_5.JPEG]
