# Supplementary material for: Acetylation of BcHpt Lysine 161 Regulates Botrytis cinerea Sensitivity to Fungicides, Multistress Adaptation and Virulence
Source: Front Microbiol. 2020 Jan 8;10:2965. doi: 10.3389/fmicb.2019.02965 (PMC6960119; doi:10.3389/fmicb.2019.02965)
Supplement: TABLE S1 — Primers used in the study. [file Table_1.DOCX]

|  | Primer | Sequence(5'-3') | Relevant characteristics |
| --- | --- | --- | --- |
| 1 | BcHpt-GFP-F | ACTCACTATAGGGCGAATTGGGTACTCAAATTGGTT TGGTTCATCTTTTTTTCTCCCTTG | PCR primers for amplification of full sequence of *HPT* gene for the subcellular localization analysis |
| 2 | BcHpt-GFP-R | CACCACCCCGGTGAACAGCTCCTCGCCCTTGCTCAC AGTGGCATAAAACTTCTTCAGGAC |  |
| 3 | BcHpt-R-F | AACCCGACAAGGAGAGATGTTTGGCGAGAA | PCR primers to amplify full *HPT* fragment including R site mutation |
| 4 | BcHpt-R-R | TTCTCGCCAAACATCTCTCCTTGTCGGGTT |  |
| 5 | BcHpt-Q-F | AACCCGACAAGGAGCAGTGTTTGGCGAGAA | PCR primers to amplify full *HPT* fragment including Q site mutation |
| 6 | BcHpt-Q-R | TTCTCGCCAAACACTGCTCCTTGTCGGGTT |  |
| 7 | BcHpt-SE | ATGTCCTCCTCTACTTCG | PCR primer for sequencing of B05.10+BcHpt^K161Q^-GFP, B05.10+BcHpt^K161R^-GFP and B05.10+ BcHpt^K161K^-GFP |
| 8 | GFP-F | TAAACGGCCACAAGTTCAG | PCR primers to amplify *GFP* fragments from B05.10+BcHpt^K161Q^-GFP, B05.10+BcHpt^K161R^-GFP and B05.10+ BcHpt^K161K^-GFP |
| 9 | GFP-R | CAGCAGGACCATGTGATC |  |
| 10 | BcHpt-up-F | AGTGACACTTGAAACAACGGG | PCR primers to amplify *HPT* upstream fragment for the construction of *HPT* deletion mutants |
| 11 | BcHpt-up-R | GACCTCCACTAGCTCCAGCCAAGCCTGACAGGCAGACTGGAAGTAA |  |
| 12 | BcHpt-down-F | ATAGAGTAGATGCCGACCGCGGGTTATCTATGGATGACGGAGGTCG | PCR primers to amplify *HPT* downstream fragment for the construction of *HPT* deletion mutants |
| 13 | BcHpt-down-R | TTCCTCGGGCCATGGAAA |  |
|  |  |  |  |
| 14 | HPH-F | GGCTTGGCTGGAGCTAGTGGAGGTC | PCR primers for amplification of the hygromycin resistant gene *HPH* |
| 15 | HPH-R | AACCCGCGGTCGGCATCTACTCTAT |  |
| 16 | BcHpt-out-F | TCCGTGCTCCAGAGTCTAAA | PCR primers for identification of *HPT* deletion transformants |
| 17 | BcHpt-out-R | TAAACCATCACCTCGTCACCT |  |
|  |  |  |  |
|  |  |  |  |
| 18 | β-tubulin-F | ACCGTTCCAGAGTTGACTCAA | PCR primers to amplify β-tubulin downstream fragment for the expression levels analysis |
| 19 | β-tubulin-R | GCAAGAAAGCCTTTCTTCTGA |  |
| 19 | BcHpt-ex-F | GGGAGTTCAGTCGTTCTATC | PCR primers to amplify *BcHPT* downstream fragment for the expression levels analysis |
| 20 | BcHpt-ex-R | CAAACATTTCTCCTTGTCGG |  |

**Table S1. Primers used in the study**
